# Artificial Intelligence in Ocular Oncology: Differentiating Choroidal Melanocytic Lesions

**DOI:** 10.1016/j.xops.2025.100948

**Published:** 2025-09-25

**Authors:** Lamis Alharby, Edward Korot, Pearse A. Keane, Sara E. Lally, Sandor Ferenczy, Lauren A. Dalvin, Marco Pellegrini, Jay Duker, Adrian T. Fung, Swathi Kaliki, Kaan Gunduz, Minoru Furuta, Antonio Yaghy, Carol Shields, Prithvi Mruthyunjaya, Mandeep S. Sagoo

**Affiliations:** 1 NIHR Biomedical Research Centre for Ophthalmology at Moorfields Eye Hospital & UCL Institute of Ophthalmology, London, UK; 2 Byers Eye Institute, Stanford University, Stanford, California; 3 Moorfields Eye Hospital & UCL Institute of Ophthalmology, London, UK; 4 Wills Eye Hospital, Philadelphia, Pennsylvania; 5 Mayo Clinic, Rochester, Minnesota; 6 Luigi Sacco Hospital - University of Milan, Milan, Italy; 7 Tufts University, Medford, Massachusetts; 8 The University of Sydney and Macquarie University Hospital, Sydney, Australia; 9 The Operation Eyesight Universal Institute for Eye Cancer, L V Prasad Eye Institute, Hyderabad, India; 10 University of Ankara, Ankara, Turkey; 11 Fukushima Medical University, Fukushima City, Japan & Tohoku University, Sendai, Japan; 12 Byers Eye Institute, Stanford, California

**Keywords:** Artificial intelligence, Choroidal melanocytic lesions, Choroidal melanoma, Choroidal nevus, Ocular oncology

## Abstract

**Purpose:**

This article explores the application of artificial intelligence (AI) in the differentiation of choroidal melanocytic lesions, specifically choroidal nevi and small melanomas, within the field of ocular oncology. The primary topic highlights the significance of accurately diagnosing these lesions to enhance patient outcomes and management strategies.

**Design:**

The study reviews of the role of AI in differentiating choroidal melanocytic lesions, particularly choroidal nevi from small melanomas, examining clinical and imaging risk factors. It explores deep learning (DL) applications for image classification and assesses AI's potential impact on patient care, diagnostic accuracy, and regulatory concerns in ocular oncology.

**Methods:**

To achieve this, the methods discussed in this paper revolve around employing DL techniques, which utilize artificial neural networks to analyze high-dimensional medical images. This approach enables automated classification and image analysis of ophthalmic data, allowing for the identification of intricate patterns and features that may be imperceptible to clinicians. Additionally, the text reviews existing clinical and imaging risk factors associated with the growth of choroidal nevi into melanoma, leveraging this information to inform and enhance AI algorithms.

**Results:**

The anticipated results of integrating AI into clinical practice include increased diagnostic accuracy, which can lead to earlier identification of high-risk lesions and, consequently, timely interventions. This proactive approach has the potential to improve patient care significantly by facilitating better management strategies, thus enhancing patient outcomes. Artificial intelligence may also uncover subtle imaging features that would otherwise be overlooked, providing a more comprehensive assessment of lesions.

**Conclusion:**

In conclusion, the paper emphasizes the transformative potential of AI in ocular oncology, advocating for its integration with existing imaging technologies. While AI offers promising advancements in diagnostic practices and patient care, the paper also acknowledges the necessity of addressing regulatory and implementation challenges to fully harness these benefits. Overall, the incorporation of AI technologies into the diagnostic workflow has the potential to not only save vision but also improve survival rates, marking a significant step forward in the management of choroidal melanocytic lesions.

**Financial Disclosure(s):**

Proprietary or commercial disclosure may be found in the Footnotes and Disclosures at the end of this article.

Choroidal nevus is the most commonly encountered intraocular lesion, found in approximately 5% to 10% of White adults, and is benign with low malignant potential.^1^ Choroidal melanoma is rare, occurring in about 2500 persons per year in the United States and about 8000 persons per year worldwide, and is fatal in approximately 40% of patients at 10 years follow-up, dependent on numerous factors, including cellular and genetic metastatic potential, age of the patient, and tumor parameters.^2^ Phenotypically, melanoma comprises a wide spectrum—from large tumors which require enucleation to small lesions with well-characterized high-risk clinical features. Both nevi and melanomas can be pigmented or nonpigmented.^1^^,^^3^^,^^4^ As even small melanomas carry malignant potential, some ocular oncology centers advocate early treatment.^1^^,^^3^^,^^5^, ^6^, ^7^, ^8^, ^9^, ^10^

There also exists a “grey area” of lesions, which can be labeled suspicious nevus, nevoma, indeterminate lesion, or small melanoma, depending on their clinical characteristics. Due to their uncertain growth potential and malignant status, long-term monitoring is usually required with multiple office visits.^10^^,^^11^ There has been a drive to use imaging to document these lesions, growth being a surrogate marker for malignant transformation.^3^

Although pigmented fundus lesions are common, and the large majority are not harmful, distinguishing small melanomas from benign nevi remains a challenge.^11^^,^^12^ A major unmet need is an easy method to differentiate suspicious choroidal nevi from small choroidal melanoma. Choroidal nevus and small choroidal melanoma can show several overlapping features, and the challenge is to identify the single small malignant melanoma among the thousands of benign choroidal nevi, which can potentially demonstrate basal diameter enlargement of 1.0 to 1.5 mm over a decade or more, representing benign growth without malignant behavior.^13^, ^14^, ^15^, ^16^ Biopsy to secure the diagnosis is often not possible or undesirable, so traditionally, observation for growth has been the paradigm for managing such indeterminate lesions. However, to improve survival, many ocular oncology centers have taken the approach of offering treatment earlier, diagnosing choroidal melanoma when small, based mainly on well-described clinical and imaging features,^17^ despite the risk to vision in some cases.

The mnemonic “To Find Small Ocular Melanoma Doing IMaging” (TFSOM-DIM) represents (T) for thickness >2 mm on ultrasonography, (F) for subretinal fluid (SRF) on OCT, (S) for symptoms of reduced Snellen visual acuity <20/50, (O) for orange pigment on autofluorescence, (M) for melanoma hollow on ultrasonography, and (DIM) for diameter >5 mm on color photography. The more risk factors, the higher the risk of growth into a melanoma.^3^^,^^13^^,^^18^, ^19^, ^20^ The recently described MOLES clinical scoring system represents Mushroom shape, Orange pigment (ie, lipofuscin), Large tumor size, Enlarging tumor, and SRF. Each of these features is scored between 0 and 2, and tumors are diagnosed according to their sum total as “common nevus,” “low-risk nevus,” “high-risk nevus,” or “probable melanoma”^5^, ^6^, ^7^^,^^21^ (Fig 1).

**Figure 1:**
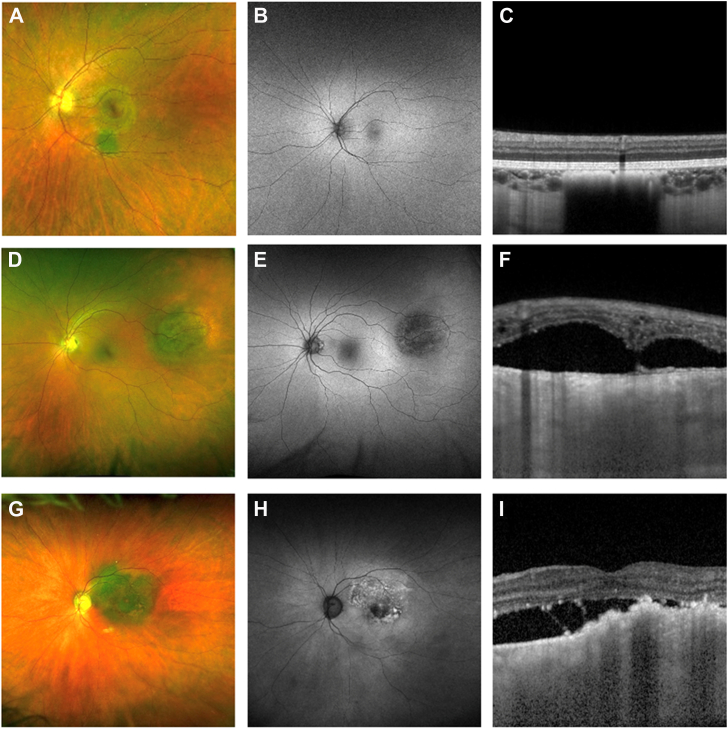
Color fundus photographs, fundus autofluorescence images, and OCTs of 3 cases: (**A–C**) choroidal nevus with basal diameter 2 mm, no SRF, and flat on USG. **D–F,** Indeterminate choroidal lesion with basal diameter 5 mm, presence of SRF, and 1.1-mm thickness on USG. **G–I,** Choroidal melanoma with basal diameter >5 mm, confluent orange pigment, presence of SRF, and 1.6-mm thickness on USG. SRF = subretinal fluid; USG = ultrasonography.

Driven by the strong interest for early, objective, and repeatable diagnosis of choroidal melanoma, algorithmic image analysis techniques have garnered interest for differentiating between these lesions. In 1956, at the Dartmouth Artificial Intelligence Conference, McCarthy et al^22^ postulated that “every aspect of learning or any other feature of intelligence can in principle be so precisely described that a machine can be made to simulate it.”^23^ Artificial intelligence (AI), which developed from this concept, describes the creation of computerized systems which perform tasks requiring synthesizing knowledge and intelligence. Ophthalmic screening is well-suited for the application of AI due to well-understood relationships between clinical features and disease severity and the ready availability of imaging to effectively capture these features. Screening programs have been extensively investigated for glaucoma and diabetic retinopathy.^17^^,^^24^, ^25^, ^26^, ^27^

Health care imaging generates large amounts of data, and >97% of it is unanalyzed or unused.^28^ Artificial intelligence will be a critical factor in managing and analyzing these data sets with the potential of doing this time-intensive work in as little as a few seconds.^26^^,^^27^^,^^29^, ^30^, ^31^, ^32^, ^33^

Deep learning (DL), a new branch of AI, has shown robust results that have surpassed human performance in many areas of health care.^25^^,^^29^^,^^30^ As a subset of machine learning (ML), DL is a highly specialized artificial neural network that consists of layers of “neurons” with activation functions forming complex neural networks inspired by the ones constituting the human brain. Similar to human learning from experience, DL algorithms are trained on a task and fine-tune based on specific examples, allowing for generalization and inference on previously unseen input. These attributes make them powerful tools for segmentation, classification, and predictive modeling.^29^^,^^34^

In February 2021, a “White Paper on Ophthalmic Imaging for Choroidal Nevus Identification and Transformation into Melanoma” was published to discuss the evolution of noninvasive diagnostic methods for identification of choroidal nevus and determination of risk factors for malignant transformation, as well as introduce the novel role that AI can play in the diagnostic process.^35^

The purpose of this paper is to discuss the potential role of AI in differentiating choroidal melanocytic lesions. We review published clinical and imaging risk factors for malignant transformation of choroidal nevus and discuss how AI applied to existing ophthalmic imaging systems may be able to determine features on medical images in an automated way. This paper did not involve the study of human participants, and therefore, no informed consent was required. Furthermore, the article adheres to the tenets of the Declaration of Helsinki.

## Summary of the Collaborative Community on Ophthalmic Imaging

As part of the Collaborative Community on Ophthalmic Imaging Conference 2020, the ocular oncology working group collaboratively presented and explored the role of AI in the diagnosis and management of intraocular tumors. The group discussed basic clinical features of choroidal nevus and melanoma, as well as imaging, and how it can be improved by using advanced techniques like swept-source OCT. It has an increased signal penetration with a greater potential to detect specific vascular patterns and can also perform a better tumor depth assessment. Machine learning using swept-source OCT could predict new features on images, discovering malignancy or malignant transformation.

With the focus on the importance of distinguishing choroidal nevus from choroidal melanoma, the group highlighted important considerations when examining patients with choroidal nevus, comprising (1) multimodal imaging risk factors which can predict risk for choroidal nevus transformation to melanoma, (2) features of chronicity (drusen, retinal pigment epithelium changes, and fibrous metaplasia) providing reassurance, and (3) change over time parameters (expansion of basal diameter, increased thickness, and new risk factors) that can indicate benign nevus growth or transformation to melanoma.

To bring ocular oncology to the next technological level, AI could be harnessed to read images and identify patients at high risk. This has been implemented in other branches of medicine and ophthalmology. A 2016 publication on AI for skin lesion classification utilizing the dermatoscope was cited, showing that AI could successfully reproduce the decision-making process of dermatologists.^36^ Other reports described the application of AI for image analysis for diabetic retinopathy, glaucoma, and age-related macular degeneration with similar successful outcomes.^37^

A good approach in ocular oncology would be first to study a very common tumor like choroidal nevus. If all nevi can be successfully identified, and if the ML model can discriminate those at risk for malignant transformation, it might be possible to treat melanoma very early or even eradicate melanoma by treating those nevi at highest risk.^38^^,^^39^

A major interest lies in the exciting opportunities of DL methodology. The continuous refinement of the model and the ability to derive independent criteria for classification, segmentation, and prediction based on the images has even greater potential. In dermatology, a DL model allowed it to grow from a classifier to an identifier. By learning from a large number of dermatoscope images, AI was able to generate its own diagnostic criteria for detecting melanoma, achieving a higher sensitivity in correctly identifying the melanoma and higher specificity in correctly identifying the nevus.^40^

It seems reasonable that applying a similar methodology to choroidal nevus holds the potential to provide beneficial results with AI, specifically with DL methodology.

Focus points for ocular oncology are to improve the detection of choroidal nevus and to find a way to obliterate a suspicious choroidal nevus or early melanoma without harming a patient's vision. Next to multimodal imaging features, AI has the potential to help with the improved detection of suspicious features. Installing such technologies in community care would provide convenient and early access to care for patients.

Despite general ophthalmologists being well-trained and capable of recognizing abnormal lesions, there are 2 barriers that impede accurate diagnosis. First is the concern of a misdiagnosis, so there can be a push to upgrade a lesion. Second, not all of the specialized imaging that is common in a tertiary level ocular oncology clinic is available in a general community clinic. Reliance on only a few modalities rather than multimodal imaging may miss important imaging features. Both factors generate over-referrals into tertiary care. Use of AI with or without remote advice from a specialist would support those in primary and secondary eye care.

Though early detection and treatment are important, an adjuvant treatment for cases with metastatic potential is important in improving patient survival. In this multifaceted approach, the potential of AI for early diagnosis is keenly anticipated. The added advantage is the cost efficiency for health care systems as well as patients with remote AI-based diagnosis. If the AI system can differentiate melanocytic lesions from other pseudotumors, its utility would be even greater.

Artificial intelligence can allow national or international standardization of protocols for diagnosis, monitoring, and even treatment. Pooled data can be used to increase the sensitivity and specificity of the system, especially with greater quantity, quality, and diversity of collected data settings. Analyzing large data sets can be computationally heavy and time-consuming, a challenge that can be overcome with the advent of novel computers with increased computing power. In addition, a robust ML model requires strong data sets that consist of data that meet certain predetermined standards, for example, the color photographs needing to be in a particular area of the fundus or using standardized camera settings.

In other fields, AI detection of cancers has been attempted. Published studies from 2020 demonstrate that AI detection of prostate cancer shows >98% sensitivity and 97% specificity. Using mammography imaging for detection of breast cancer looking at mammography, the AI was 95% sensitive, whereas radiologists were about 81% sensitive.^41^^,^^42^ In ocular oncology, the initial aim of AI in melanoma detection is to achieve ≥99% sensitivity, even if AI is less specific. To reach the highest possible sensitivity and specificity, many image combinations with a DL model might be necessary.

It will be challenging to develop an AI model on a limited number of photographs, especially if these have been captured by potentially 6 different modalities on the same patients. Multicenter efforts to use images not only for choroidal nevus and melanoma but also for indeterminate cases would be a critical factor for a DL model to be accurate. The barrier to this is the varying camera and imaging systems used in different clinics.

Using the multimodal approach could optimize the advancement of DL models to uncover features that practitioners cannot see with their own viewing of images or clinically. There might be inconspicuous information on OCT, fundus photography, autofluorescence, and other modality imaging that help to detect melanoma earlier.

Modern AI-assisted diagnosis of pigmented choroidal lesions can be significantly enhanced through advanced ophthalmic imaging, supported by select genetic and systemic data. Besides traditional photography, other core imaging modalities include ultrasound B-scan, which assesses lesion thickness, shape, and internal reflectivity—key features for differentiating choroidal nevi from melanomas. OCT reveals retinal and retinal pigment epithelium changes, such as SRF or photoreceptor disruption, while fundus autofluorescence highlights lipofuscin accumulation, a marker of malignancy risk. Ultrasound biomicroscopy aids in evaluating anterior extension of peripheral lesions, and magnetic resonance imaging can be useful in assessing extrascleral spread or orbital involvement in advanced cases. Although not routinely used, genetic markers such as BAP1 mutations in uveal melanoma can also provide valuable prognostic insight. Integrating these imaging and genetic inputs into a multimodal AI system supports accurate classification, risk stratification, and timely management of pigmented choroidal lesions.^43^

## Society and Patient Perspective

Artificial intelligence could potentially play a significant role in supporting the diagnosis and treatment of ocular melanoma, but how do patients perceive the use of AI as part of their health care?

A study for skin cancer screening in March 2020^44^ showed that 75% of the patients stated that they would recommend AI to friends and family members, indicating the increased diagnostic speed associated with early skin cancer detection and lifesaving potential. In general, many perceived AI benefits including the prospect of increased health care access, also supported by remote diagnosis, reduced health care costs, increased triage efficiency, and even unburdening of the health care system.

However, 94% of patients in that study expressed the importance of symbiosis between physicians and AI. Some patients pointed out the potential risk of human loss of social interaction, patient loss of privacy, patient loss to follow-up, nefarious use of AI, and human deskilling. Others also anticipate a lack of verbal and nonverbal communication and emotion. There were concerns about the inability of AI to answer follow-up questions, discuss treatment options, educate and reassure the patient, and reduce anxiety.

Another key finding of that study was the split perception concerning the accuracy of diagnosis. While 69% perceived more accurate diagnosis as the greatest strength of AI compared with humans due to the ability of AI to draw on more data or experience than humans, learn, evolve, and share data; 85% perceived less accurate diagnosis as the greatest weakness of AI, based on the potential for false-negatives and false-positives. Outside the clinical scope where credibility is crucial to trust the AI, the study also revealed that AI could have the strength to drive patient activation to seek health information and patient education.^44^

Another study from June 2019 examining patients' views of wearable devices and AI in health care supports the notion that most patients were ready to accept the use of AI but only under human control and if the integrity of the human physician-patient relationship is preserved.^45^

Though there is some room for extrapolation, additional research specific for AI acceptance by ophthalmic patients is required. At the Collaborative Community on Ophthalmic Imaging conference 2020, the impact of ocular melanoma on patients and their families was discussed, realizing that there are just a few centers around the world that offer expertise in this rare disease and that there is the need to put more effort into building accessible resources for the greater patient community. Recently, large language models for patient information in uveal melanoma have been assessed.^46^ The use of AI could be a vital aspect to back the mission of newly established initiatives, to support research and accelerate the development of effective treatments, and ultimately, a cure for ocular melanoma through innovative strategies, including international and interdisciplinary collaborations to improve the lives of people affected by ocular melanoma, by creating systems and programs to provide education and support and to advocate for the ocular melanoma community.

## Regulatory Concerns in the United States, Europe, and Globally

In many countries, regulatory approval is required before AI and ML algorithms are allowed to be used for clinical care. This is to limit the potential for harm, ensure confidentiality, and mitigate risks.^41^

At the end of 2016, the 21st Century Cures Act^47^ in the United States defined the scope of US Food and Drug Administration (FDA) regulatory jurisdiction over software use in health care, specifying that a medical device is an instrument or other tool intended for use in the diagnosis of disease or other conditions, or in the cure, mitigation, treatment, or prevention of disease, in man or other animals, or intended to affect the structure or any function of the body of man or other animals.^48^ Every AI system falling within this definition will be regulated by the FDA, as provided by the Federal Food, Drug, and Cosmetic Act.^49^ The FDA categorizes medical devices into 3 classes, according to their uses and risks, and regulates them accordingly, where class III is the category that includes the devices involving the most significant risk. In recent years, companies using AI-based clinical support software applications and medical devices, for example, for analyzing computed tomography angiogram images or diabetic retinopathy screening, have requested de novo classification from the FDA. This alternate pathway of FDA regulatory approval applies to novel medical devices whose type the FDA has not previously classified, and which allows a more streamlined risk-based classification based on reasonable assurances of safety and efficacy.

The FDA also initiated the Expedited Access Pathway and established another category of so-called “breakthrough devices” for medical devices that demonstrate the potential to address unmet medical needs for life-threatening or irreversibly debilitating diseases or conditions. Under the Expedited Access Pathway, the FDA works with device sponsors to reduce the time and cost from development to marketing decision.^50^

The black box nature and the rapid growth of AI/ML applications, including the volume and complex nature of testing and verification, are challenging factors for the FDA to approve the numerous new medical devices and algorithms that are continuously being developed. As of November 2024, there are >900 AI/ML-based FDA approved medical devices and algorithms, with the number of approvals constantly increasing and spanning across various medical specialties such as Radiology, Cardiology, Internal Medicine, and Ophthalmology.^51^, ^52^, ^53^, ^54^

The FDA's 2021 paper, “Artificial Intelligence and Machine Learning (AL/ML) Software as a Medical Device Action Plan” acknowledged the unique challenges in regulating AI systems and proposed a new regulatory framework in which adaptive algorithms may not need to be reapproved.^47^

In January 2025, the US Government issued the order “Removing Barriers to American Leadership in Artificial Intelligence” which aims to maintain and enhance the United States' position as a global leader in AI. It emphasizes the importance of developing unbiased AI systems and includes a review and potential rescission of previous policies that hinder innovation. Overall, the order seeks to promote a conducive environment for AI innovation while addressing previous barriers.^55^

The potential impact on health care could mean that medical AI development is progressing with fewer regulations, leading to quicker implementation by health care providers. However, this raises concerns about patient safety and fair treatment, as there are no longer requirements for bias testing or safety validation. Advocacy groups warn that this lack of safeguards could negatively impact patient care, particularly in terms of health care equity.^56^^,^^57^

The US Health Insurance Portability and Accountability Act of 1996 is another central compliance focus,^58^ which defines the use of personally identifiable health information for AI development with the purpose to prevent harms related to privacy and security.^59^ The majority of AI systems require extensive data sets for training and validation, and in clinical settings, these usually originate from aggregated personal health information. Repurposing personal health information for use in establishing an AI system is a secondary use. Many patients are not aware of common secondary uses of their health data and biological material. Consent for data collection does not ensure continuing consent for other purposes.^60^ In medicine, health care data sets usually are deidentified before being used by developers. Under the Health Insurance Portability and Accountability Act of 1996, a data set where all identifiers have been removed is no longer subject to Health Insurance Portability and Accountability Act of 1996 privacy restrictions.^58^

In 2018, the American Medical Association released a policy report on clinical AI. It included the principles that clinical AI should demonstrate best clinical practices, be reproducible and transparent, avoid disparities and bias, and protect patients' privacy.^61^

The European Union is reforming AI regulation in the context of medical device development with new legislation such as the General Data Protection Regulation (GDPR),^62^ Cybersecurity Directive, and Medical Devices Regulation/In Vitro Diagnostic Medical Device Regulation. This reform will be realized gradually with the GDPR and the Cybersecurity Directive having taken effect in May 2018.

The GDPR includes a right to an explanation of automated decisions.^63^ Article 22(3) GDPR holds that, in certain cases of automated processing, the data controller ought to implement acceptable measures to safeguard the individual's data rights and freedoms and legitimate interests.

In July 2016, the European Union adopted the “Cyber Security” Directive on security of network and information systems (2016/1148) (“NIS Directive”). Digital service providers and companies that operate essential services must protect their information technology systems and inform security incidents to the appropriate regulatory body.^64^

In the European Union and United Kingdom, the definition of medical device is provided by Article 1(2) of Directive 93/42/European Economic Community.^62^^,^^65^ The European Commission has also published guidelines to interpret requirements set out in the Medical Devices Directive.^66^ The Medical Devices Directives are nonlegally binding guidelines to guide stakeholders in complying with legislation related to medical devices.^67^

Depending on its functionality, the term medical device is applied to any instrument or tool, including any kind of software, intended by the manufacturer to be used for human beings for the purpose, among others, of diagnosis, prevention, monitoring, treatment, or alleviation of disease. This definition requires manufacturers to ensure that the devices they produce are fit for their intended purpose.^67^, ^68^, ^69^ This regulatory framework has been reformed by the new Medical Devices Regulation^69^ and the new In Vitro Diagnostic Medical Device Regulation, which both came into force on May 25, 2017.^67^

In the United Kingdom, the United Kingdom National Health Service has issued a “Code of Conduct for Data-Driven Health and Care Technologies,” of which the principles consist of comprehending persons' needs, distinctly specifying the expected outcomes and benefits, lawful data processing, transparency, and evidence of effectiveness and safety.^70^ National Health Service England's Long Term Plan sets the direction toward widespread digitally enabled care with a clear ambition for the generation of more digital services designed around user need and adhering to key principles of privacy, security, interoperability, and inclusion.^70^ The National Health Service AI Lab aims to bring together policies, partners, and programs to develop and deploy safe, effective AI applications.^71^

In 2019, the World Health Organization (WHO) started developing a framework for the adoption of digital innovations and technology in health care. The WHO recommendations on digital interventions in health care promote assessment on the basis of “benefits, harms, acceptability, feasibility, resource use, and equity considerations.”^38^ Digital technologies in the health care sector are increasingly adopted by the WHO member States, investing in the use of data for decision-making and considering modern solutions to empower their health systems.^72^

The WHO has made a commitment to address ethics, governance and regulation of AI for health and, in 2019, established an expert group to help develop a global framework for ethics and governance in AI. The goal of this initiative is to ensure that these technologies are aligned with the overarching aims of promoting fair and equitable global health, meeting human rights standards, and supporting member States' commitments to achieve universal health coverage.^73^

## Benefits and Challenges of AI in Ophthalmic Imaging and Diagnosis

Undoubtedly, AI is a tool with enormous potential. Algorithms can integrate complex information faster and more reproducibly than humans.^74^ A big focus of the implementation of AI in the health care sector lies in medical imaging, automated clinical decision-making, diagnosis, and prognosis.

Artificial intelligence has the potential to improve the current landscape of imaging systems in ≥3 ways. First, it can help in terms of time efficiency and speed up processes. Artificial intelligence systems can effectively recognize subtleties in visual images that might suggest various disease scenarios in near real-time.^75^ Integrating AI into hospitals and clinical settings will improve the efficiency of the health care system. It will save time, reduce the workload and documentation burdens for physicians, and allow them to concentrate on tasks that computers cannot perform, such as enhancing patient interactions.^76^^,^^77^

Where AI is used for imaging systems, AI-driven image analysis software will allow specialists such as ophthalmologists, who commonly analyze images, to spend less time screening and instead concentrate on diagnosis and decision-making. The same technology will provide nonspecialized physicians with digital assistance to interpret medical images, making them less reliant on specialized hospital departments.^78^

Second, AI may help in terms of cost. It is expensive to hire professionals to read medical images, and when having limited resources, it is important to employ them as efficiently as possible. Artificial intelligence can accomplish accurate image analysis in a cost-efficient manner.

The third benefit of AI is diagnostic performance. Numerous studies have revealed evidence that AI can grade images as accurately or potentially even more accurately than professional graders. For example, one criterion for intermediate-stage or moderate nonproliferative diabetic retinopathy can involve counting the number of hemorrhages or other features in the image indicating different stages of the disease. If a human is conducting this task, it can be very time-consuming and not necessarily more accurate than if a machine does the analysis.^25^, ^26^, ^27^^,^^29^^,^^30^^,^^33^^,^^79^ Studies have also shown successful results in detecting and differentiating malignancy with high accuracy by using AI for image analysis.^80^, ^81^, ^82^

Furthermore, by deploying DL methodologies to advance AI-based ophthalmic image analysis, AI may possibly uncover associations between disease and detectable characteristics in the eye that doctors are currently unaware of. By scanning patterns among millions of pixels per image, comprised in data sets of millions of patients, that will never be matched by ophthalmologists, DL will perhaps detect new features outside the well-known risk factors. It might also be capable of correlating to other ophthalmic clinical characteristics and predict the patient's individual risk for certain diseases.^83^^,^^84^

Artificial intelligence may not only provide information about the patient's existing disease state but furthermore might present insights into the pathophysiology of diseases through discovering novel features.^24^^,^^74^

An example is RETFound, a recently developed foundation model for retinal images that learns generalizable representations from unlabeled retinal images and provides a basis for label-efficient model adaptation in several applications. Trained on 1.6 million unlabeled retinal images through self-supervised learning, it adapted for disease detection tasks with explicit labels. This approach enhanced the model's ability to adapt to specific disease detection tasks using fewer labeled data. RETFound significantly outperforms other models in diagnosing sight-threatening eye diseases and predicting systemic disorders such as heart failure and myocardial infarction.^85^

In 2023, Wagner et al investigated inner retinal anatomy, measured using OCT, in prevalent Parkinson disease and subsequently assessed the association of these markers with the development of Parkinson disease using a prospective research cohort. It was discovered that individuals with Parkinson disease have reduced thickness of the inner nuclear layer and ganglion cell-inner plexiform layer of the retina, and that involvement of these layers several years before clinical presentation highlights a potential role for retinal imaging for at-risk stratification of Parkinson disease.^86^

To reap the full potential of AI and ML's significant benefits and opportunities, organizations must mitigate the considerable risks these innovations introduce. Legal issues, data privacy, and security concerns are some of the biggest challenges, with a specific caveat when evaluating AI use in health care. Artificial intelligence applications with an incorrect algorithm and an ill-defined data governance strategy can cause significant legal challenges for an organization and its services.

Computing power can also be a challenge. Powerful algorithms utilized for ML and DL methods require an ever-increasing computational power to execute these algorithms successfully. Cloud computing and parallel processing systems allow developers to work with AI systems more efficiently; however, the increase of extraordinary amounts of data and the development of increasingly complex algorithms pose a real burden. Furthermore, resource-intense computing methods negatively impact the environment through high energy consumption, resource extraction for hardware production, and increasing electronic waste. To mitigate these effects, adopting sustainable practices in computing is essential.^87^

Besides, there is still limited knowledge and a scarcity of field specialists in AI.^88^ The development of AI technologies requires specialists who need to undergo a complex and demanding education to acquire the relevant knowledge, represent it, and utilize it appropriately to create models and explain the findings. Artificial intelligence specialists are expensive and desired in many sectors and industries.^34^^,^^89^

Ethical concerns will become apparent as AI can be applied to almost any field in medicine, and its potential contribution to health care seems limitless.^90^ Scientific and academic institutions play a key role in promoting and supporting ethical AI. Increasing accuracy, minimizing bias, maximizing generalizability, broadening utility, assuring safety, enhancing transparency, and ensuring accountability are all ethical obligations and are complementary to strive for autonomy, beneficence, and justice.

The culture of medical institutions is often slow to accept change in working practices. Physicians may show concern regarding the loss of jobs as AI becomes more integrated; in one study, 14% were worried about the loss of their jobs and 30% regarding the loss of professional skills.^90^ Image intensive specialties such as radiology could even be threatened by AI, though the human interpersonal interaction in ophthalmology makes it less vulnerable.^24^^,^^74^ The concern that AI could cause ophthalmologists' skills to deteriorate is counterbalanced by the argument that AI is a new way to address the global shortage of health care professionals.^74^^,^^91^^,^^92^

Explainability and trust deficits are emerging issues in AI. Over time, humans entrust more decisions to ever more complex computer systems, and there is a tendency to overtrust automated decision-making systems.^93^, ^94^, ^95^

Some scientists caution that there is a threat in relying on AI systems for explainability and consistency, and we ought to acknowledge unpredictability and variance of outcomes. On the other hand, AI systems are often provided as being “black boxes,” that are difficult to explain. This black box problem presents concerns for users who lack insight into what the AI is, in fact, doing, and they are worried about basing decisions on information they cannot see. Given how mathematical modeling works in these AI systems, it is difficult to detect what the decision tree looks like. When a user, such as an ophthalmologist, does not understand that a new feature in the image leads to a clinical diagnosis of choroidal melanoma, they may be skeptical. In an ideal scenario, the ophthalmologist could interrogate the black box and see how AI came to its response, and that access might also provide physicians information from which they can learn. Due to the inability to interrogate a DL model, some caution should be exercised when making assumptions on a model's generalizability. For example, an algorithm could incorrectly form associations with confounding nonpathological features in an image (such as imaging device, acquisition protocol, or hospital label) simply due to differences in disease prevalence in relation to those parameters.^96^

Explainability is an intensely debated topic, specifically when it comes to the application of AI in health care.^97^ There are alternative approaches to so-called “Intelligible Machine Learning,” which eliminate some of the downsides of the “black box.” This refers to systems that are able to describe what they have learned, which would be very significant for AI use in health care. If an AI system produces an error, it may be problematic to identify the precise place where the fault occurred within the “black box” like system. Scientists succeeded in developing methods for “white box” testing for DL systems.^33^ This tests neural networks with a large number of inputs and detects where their responses are incorrect so they can be rectified. Other methods include the generation of saliency maps that help pinpoint which areas in an image the AI focused on in its decision-making process.

Data scarcity and quality are important considerations, as having enough data is a critical element of AI. Usually, a high number of labeled data, for example, labeled images, is used to train machines to learn effectively and make accurate predictions. Several studies have proven that more data leads to better model performance.^79^^,^^98^ In the publication by Shields et al,^35^ image labeling techniques and various types of AI learning approaches, such as supervised learning and DL, are described in detail, with special consideration for choroidal lesions. To train an AI model for image classification aimed at distinguishing melanocytic lesions, the gold standard would be a carefully curated data set of images categorized into 3 classes: nevus, indeterminate, and melanoma, and meticulously labeled by experts based on established grading guidelines, such as TFSOM-DIM or the Mushroom shape, Orange pigment (ie, lipofuscin), Large tumor size, Enlarging tumor, and SRF scoring system. A consensus grading system solely for AI use may be necessary to improve the categorization into the clinical diagnostic classes. Using this gold standard allows the model to learn the correct patterns associated with clinical features and risk factors for malignant transformation, as well as their relationships within the data, resulting in more reliable predictions.

Different hospitals and services handle tumor samples with various instruments and protocols. Teaching one algorithm to navigate all that variability will be a steep task, and training the AI model with more data from more varied sources is essential for achieving near-perfect reliability.^98^ There are further considerations on how the data should be labeled for the AI model to learn and on how to feed acquired knowledge back into the system. To improve the effectiveness and accuracy of the AI model, generating a feedback loop to continuously enhance the model on the basis of people's actions and decisions is an important step.^99^

The need for suitable training data and external verification is a technical challenge that needs to be addressed to facilitate generalizability and translation of AI solutions.^100^ A recommendation would consist of categorizing the data sets associated with the development of an algorithm into 3 sets: a training set (for training the algorithm), a validation set (for tuning hyperparameters which are set before the learning process and used to optimize model training), and a test set (for estimating the performance of a final tuned model when comparing it to other final models). It is crucial that the test set contains data independent to training or validation data and are used only for assessing the final model. To define the different types of test sets, the guidance by Altman and Royston can be followed to perform internal validation, temporal validation, and external validation.^101^

Artificial intelligence bias is an irregularity in the output of ML algorithms and may occur due to prejudiced assumptions made during the formation and development of the algorithm or bias in the training data. Literature shows that AI-based systems applied in health care have flaws that adversely impact their capability to perform at an expected level. Three parameters can be recognized that result in a bias in the knowledge base of an AI system^102^: (1) bias due to inaccurate data analysis, (2) bias due to incorrect information derived from a credible resource, and (3) bias due to experimental design and implementation. Artificial intelligence systems may also contain biases due to cognitive biases and lack of complete data.^103^

A difficulty with ML models is that there are always imperfections in the data set used to train the model. Data sets may include or omit certain specifications, with the consequence that the ML model learns based on flawed data, and the output may impact patient safety. Performance on artificial data sets may be different from natural scenarios, due to different testing conditions, such as lighting, software, and imaging variables, as well as patient variables. Artificial intelligence image analysis can be confused by parts of an image that would not influence a human, an unintentional bias. Deep learning systems may draw false conclusions based on information we would not want them to include. The consequence could be a risk for patient safety.

Artificial intelligence is sometimes unintentionally used on a diverse population from the one on which it was trained, and it would be unsuitable for a model to be applied to a population that differs in crucial ways from the learning data set. The same would be valid for patient demographics. Because AI decisions are a direct reflection of its input data, the data it acquires must have an accurate representation of patient demographics; for example, White males are overly represented in medical data sets.^42^ Training on minimal minority data may cause the AI to generate more accurate predictions for majority populations, resulting in unintended poor medical outcomes for minorities.^104^, ^105^, ^106^

To make an appropriate diagnostic decision for choroidal nevus, indeterminate lesion or a choroidal melanoma based on the pigmentation of the eye, considering the differences in demographics will be significant. Therefore, an AI algorithm trained on images collected from only a specific part of the population may introduce bias, and potentially limit its use for other parts of the population.

Another bias could be introduced by taking images with a particular imaging device using a unique setting, limiting the algorithm's ability to be applied to a broad range of different imaging devices.

There could also be a fear that AI might miss a sign, such as a risk factor of malignancy, if it is not looking for it, giving ophthalmologists and patients a false sense of security. Findings from studies comparing performance on internal versus external validation revealed that, as expected, internal validation overestimates diagnostic accuracy in both health care professionals and DL algorithms. This finding highlights the need for out-of-sample external validation in all predictive models.^107^

## Approach of Implementing AI in Ocular Oncology

The implementation of AI needs a clear strategy. Initial framing should include intimately understanding the use case, considering potential solutions, and specifying how AI could help develop a practical solution to the defined problem. It is also essential to gather appropriate knowledge and manage expectations regarding what AI can and cannot yet do for the organization and their services.

As a fundamental integration challenge, data infrastructure requirements and data storage will need to be considered, along with how the data should be labeled and fed into the system. To develop the effectiveness of the AI model, a strategy needs to be determined on how the model will be trained and tested in the best way.^99^ Dataset curation should adopt a use-case-first approach to ensure that the training data aligns with practical implementation.

**Figure 2:**
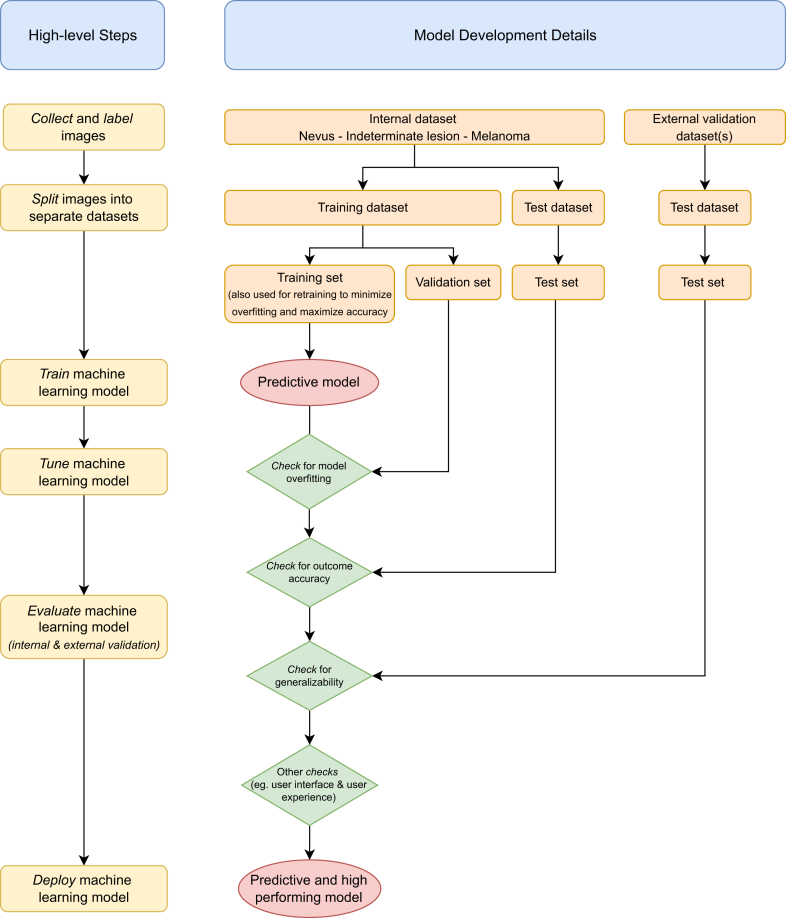
Flowchart depicting the development of AI models for distinguishing choroidal melanocytic lesions. AI = artificial intelligence.

Taking into consideration the key challenges for developing an AI model as outlined in the previous section, Figure 2 shows a suggested flow of how to develop, train, validate, test, and deploy a potential AI model with the purpose of distinguishing choroidal nevi, indeterminate lesions, and choroidal melanomas. The final model would then need to be integrated into the existing information technology infrastructure of the clinic or into the community services to serve as decision support for diagnostic and subsequent referral action.

Workflow integration and user experience are important factors for acceptance. If the AI system is unwieldy to access and not a natural part of the work the ophthalmologist is doing, it will be difficult to adopt and utilize. In addition, workflow integration helps to minimize the cognitive load. For example, if the AI system is presented in the workflow concurrent with the patient's visit, it reduces the additional effort for the ophthalmologist, such as accessing another tool to review the AI decision and then going back and updating the documentation in the electronic patient record.^108^

The field of “computer-human interaction” is concerned with how to design interfaces and realize interactions between computers and humans. The computer-human interface can be described as the point of communication between the computer and the human user. The flow of information between the computer and the human is defined as the loop of interaction. Unexpected problems can occur if these interfaces are poorly designed. One of the best practices in many successful AI deployments is termed “human-in-the-loop” computing. The AI model takes a first pass on analyzing the data, an image or document that needs labeling, and assigns a confidence score on how sure the algorithm is making the appropriate judgment. If the confidence score is lower than an accepted certain value, it circulates the data to a human expert to make a final decision. That new human judgment is used for both the clinical process as well as fed back into the AI algorithm to improve its clinical accuracy. This hybrid approach combines the best of human intelligence and AI to create a collective superintelligence that could enhance applications to work most reliably.^109^, ^110^, ^111^

Another major question is: how can AI be responsibly applied to ensure that physicians can trust the information presented? One principle is content transparency. To reduce the black box phenomenon and to focus on building trust, the AI algorithm should be as interpretable as possible, so ophthalmologists fundamentally understand how it arrived at a specific diagnostic decision of a choroidal nevus, an indeterminate lesion, or a choroidal melanoma. In practice, text displaying the rationale for each decision may be helpful, so the ophthalmologist understands at the time when they are making the final decisions what feature and clinical evidence was found.

Proving value is another step to support trust in the AI system by giving the end-user the opportunity to run a test drive of the product in a sandbox environment before making the decision to use it in the real clinic setting.^108^

The trust deficit can further be managed with thorough education, demonstration, extensive validation, and general communication of the many benefits of AI investment. Applying ML systems to guide users toward clinical decisions could improve quality measures but not automatically reflect improved care. The installation of ML in complex care practices will require ongoing audits of diagnoses to complete cycles of learning to improve diagnostic accuracy. Integrating a unique diagnosis or method of practice into an algorithm too early might indicate authenticity that is not supported by the data.^112^

Clinical validation is, hence, a key aspect of managing trust in this area. It ensures that the appropriate clinical evidence is found for every documented diagnosis and also ensures that it was present and treated if it has been documented. When starting to implement the AI system in ophthalmic practice, it would be an option to let the ophthalmologist always make the final medical decision by providing actions to either accept or dismiss an AI decision and give an opportunity to even select a different diagnosis. “Human-in-the-loop” computing would allow leveraging the advantages of the AI model with an ophthalmology expert at certain checkpoints to fill gaps where the algorithm is not yet confident or where it may fall short due to potential underlying biases.

A cautious approach applying AI in a clinical setting may prevent a model from being deployed without full understanding of risks involved. A bad outcome could significantly set back the entire field. When deploying an AI learning system to distinguish choroidal melanocytic lesions, it will be important to have substantial monitoring and quality control in place. In this way, potential mistakes or wrong decisions, such as a missed detection of a risk factor of growth, could be recognized quickly, and appropriate actions could be taken to mitigate patient safety risk. Once AI is deployed on a broader scale, there will still be a period where we learn and refine. Updated knowledge in clinical and diagnostic areas could mean that the accuracy of the model can drift, so the algorithm is no longer accurate, which can potentially lead to overprediction or underprediction. A reasonable algorithm change control would be required, which ensures validation of its reliability, credibility, and applicability.

## Conclusion

To overcome the variety of challenges outlined in this paper, the Collaborative Community on Ophthalmic Imaging ocular oncology working group was unanimous in discussing the principal approach to use AI in ocular oncology for differentiating choroidal melanocytic lesions. The goals include utilizing multimodal imaging, identifying a unique standard that everyone can use, and, most importantly, working collaboratively and internationally together to collect a pool of relevant images from a variety of populations. This may lead to robust algorithms, which could be used in various clinical settings and diverse populations. Consequently, the AI model may then successfully differentiate not only choroidal nevus from small choroidal melanoma, but also distinguish indeterminate lesions. There are emerging reports of DL algorithms to distinguish nevus from small melanoma,^39^^,^^113^ as well as predict malignant transformation.^114^ Successful implementation could lead to AI being extended to other tumor diagnoses as well as to histopathological evaluations of the specimens generated by ocular oncologists.

In ophthalmology and ocular oncology, we are beginning to see that AI may make fundamental contributions toward the provision of high-quality and sustainable eye care. Nevertheless, challenges associated with implementing these technologies remain, including validation, trust, and patient acceptance, as well as education and training of end-users on these technologies. Ophthalmologists must continue to adapt to the changing models of care delivery and collaborate with broader teams, including technology experts and data scientists, to achieve universal quality and sustainable ophthalmic services.

## Disclaimer

This article reflects the consensus views of the writing group and does not necessarily represent the practices, policies, requirements, or recommendations of the FDA. While the FDA participates in the Collaborative Community on Ophthalmic Imaging (CCOI), the FDA members have not contributed to this manuscript. Further, the use of the word “required,” “must,” or “should” in the document is not intended to indicate an FDA requirement.
